# Supplementary figures and images for: Overuse of computed tomography for mild head injury: A systematic review and meta-analysis
Source: PLoS One. 2024 Jan 11;19(1):e0293558. doi: 10.1371/journal.pone.0293558 (PMC10783716; doi:10.1371/journal.pone.0293558)

**
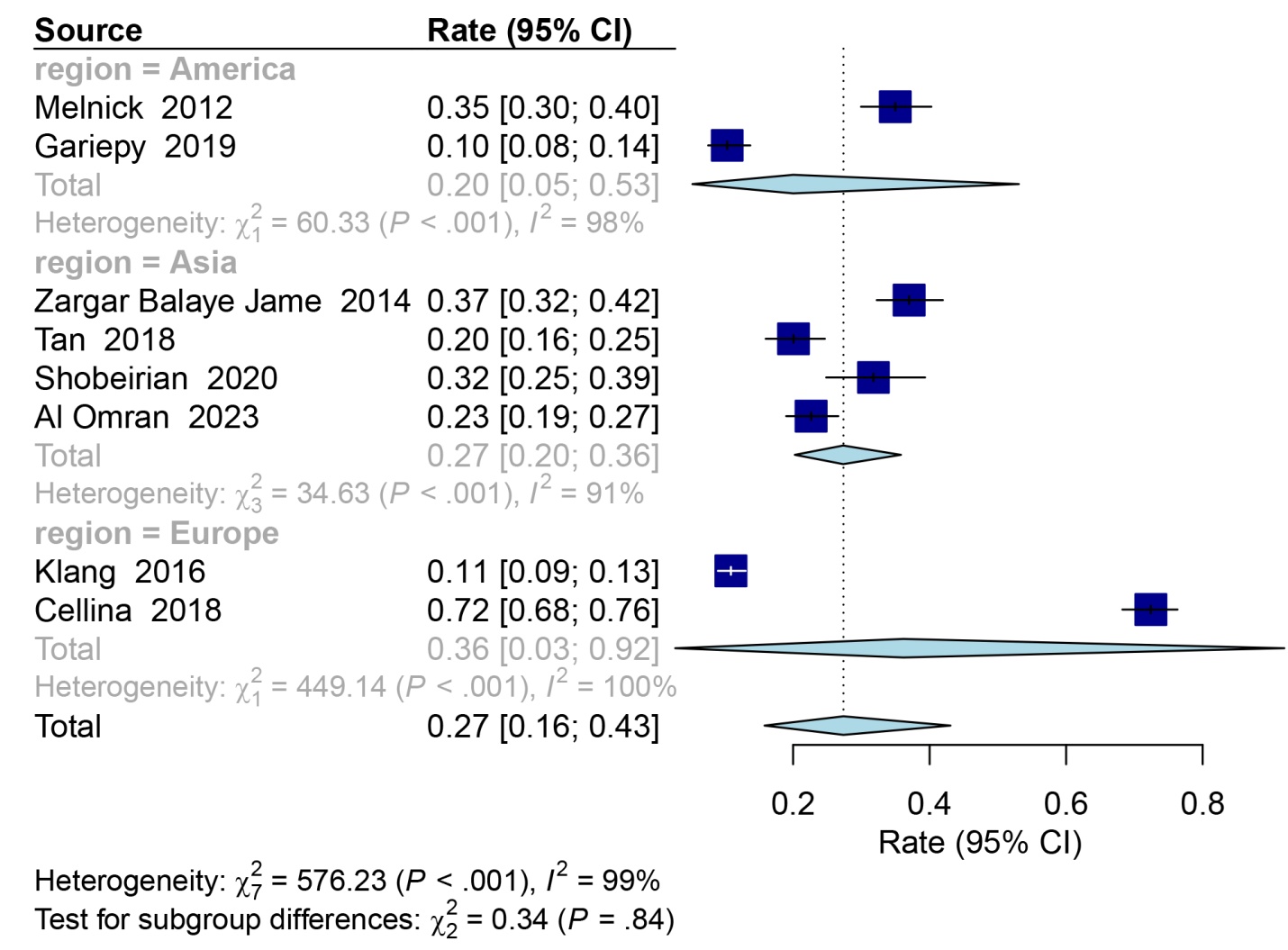
**

**S1 Fig 1.**

Supplement: S1 Fig — Random effects model used for analysis (95% confidence interval). The rate of CT overuse in mild head injury patients was 36% [95% CI: 3–92; I2 = 100%] in Europe, 27% [95% CI: 20–36; I2 = 91%] in Asia, and 20% [95% CI: 5–53; I2 = 98%] in America. (DOCX) [file pone.0293558.s001.docx]

**
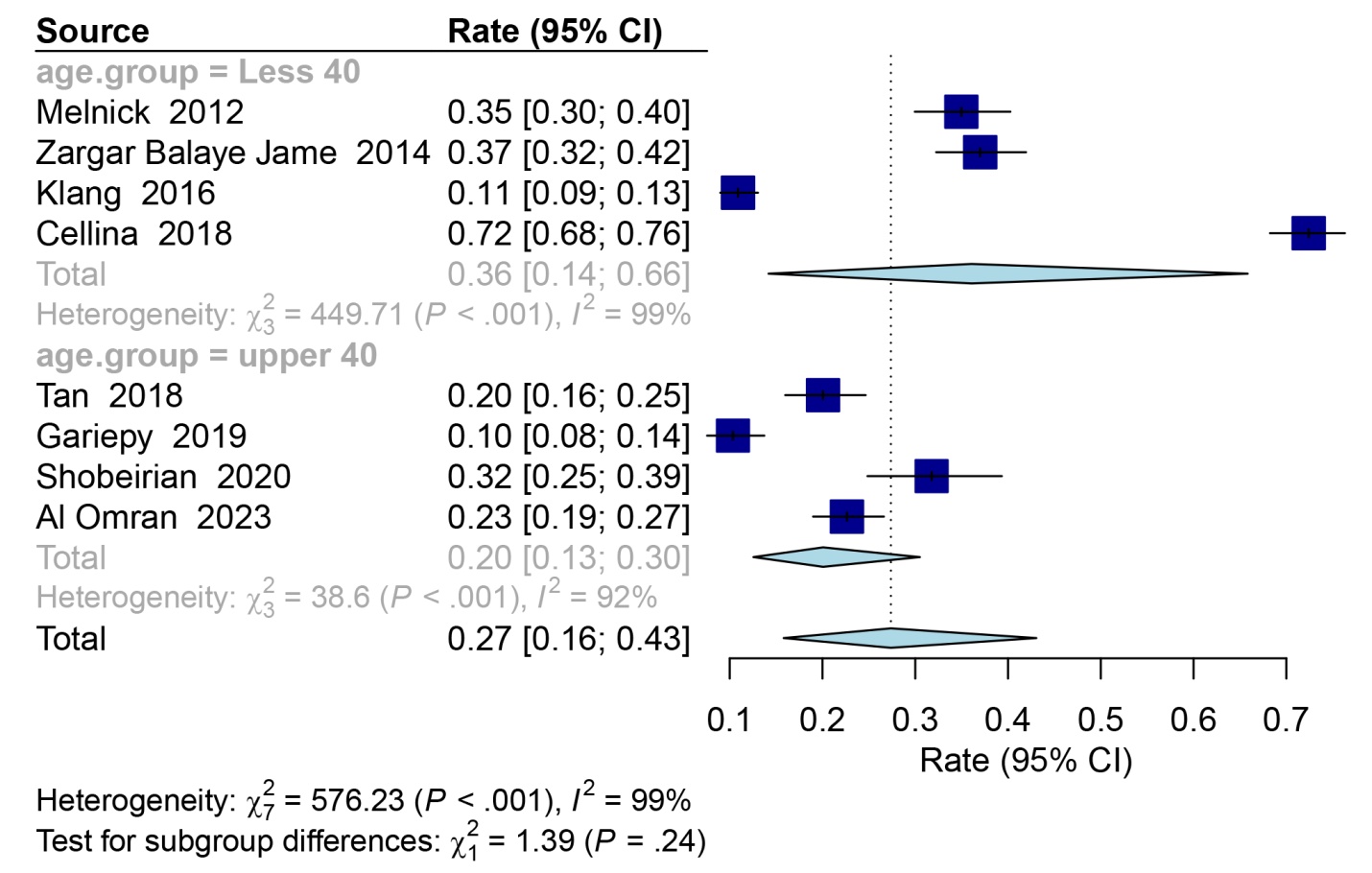
**

**S1 Fig 2.**

Supplement: S2 Fig — Random effects model used for analysis (95% confidence interval). The rate of CT overuse in mild head injury patients was 36% [95% CI: 14–66; I2 = 99%] in individuals aged 40 years and below, and 20% [95% CI: 13–30; I2 = 92%] in those above 40 years. (DOCX) [file pone.0293558.s002.docx]

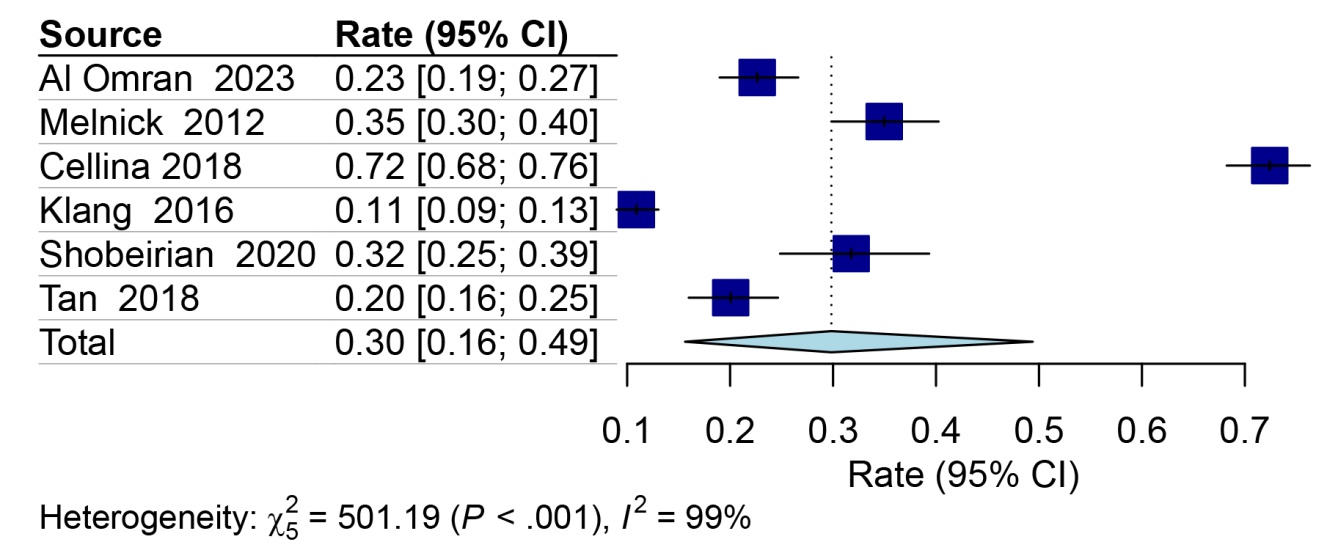


**S1 Fig 3.**

Supplement: S3 Fig — Random effects model used for analysis (95% confidence interval). The overall rate of CT overuse in mild head injury according to the CCHR was estimated to be 30% [95% CI: 16–49; I2 = 99%]. (DOCX) [file pone.0293558.s003.docx]

**
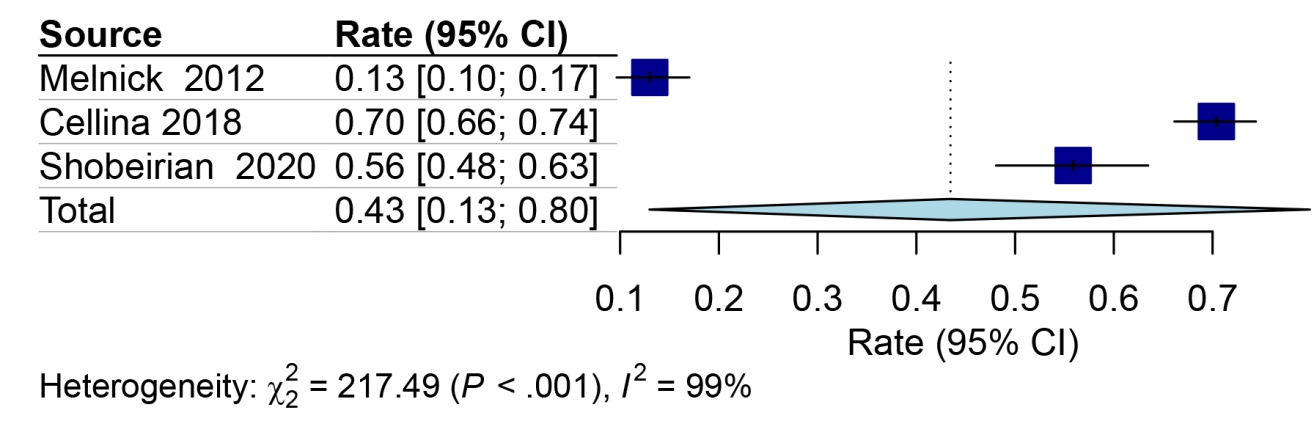
**

**S1 Fig 4.**

Supplement: S4 Fig — Random effects model used for analysis (95% confidence interval). The overall rate of CT overuse in mild head injury according to the NICE was estimated to be 43% [95% CI: 13–80; I2 = 99%]. (DOCX) [file pone.0293558.s004.docx]

**
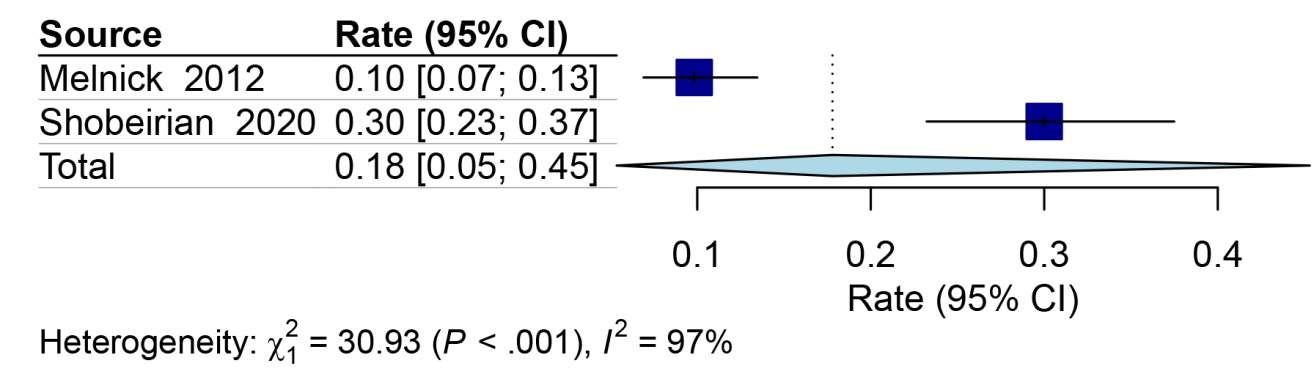
**

**S1 Fig 5.**

Supplement: S5 Fig — Random effects model used for analysis (95% confidence interval). The overall rate of CT overuse in mild head injury according to the NOC was estimated to be 18% [95% CI: 5–18; I2 = 97%]. (DOCX) [file pone.0293558.s005.docx]

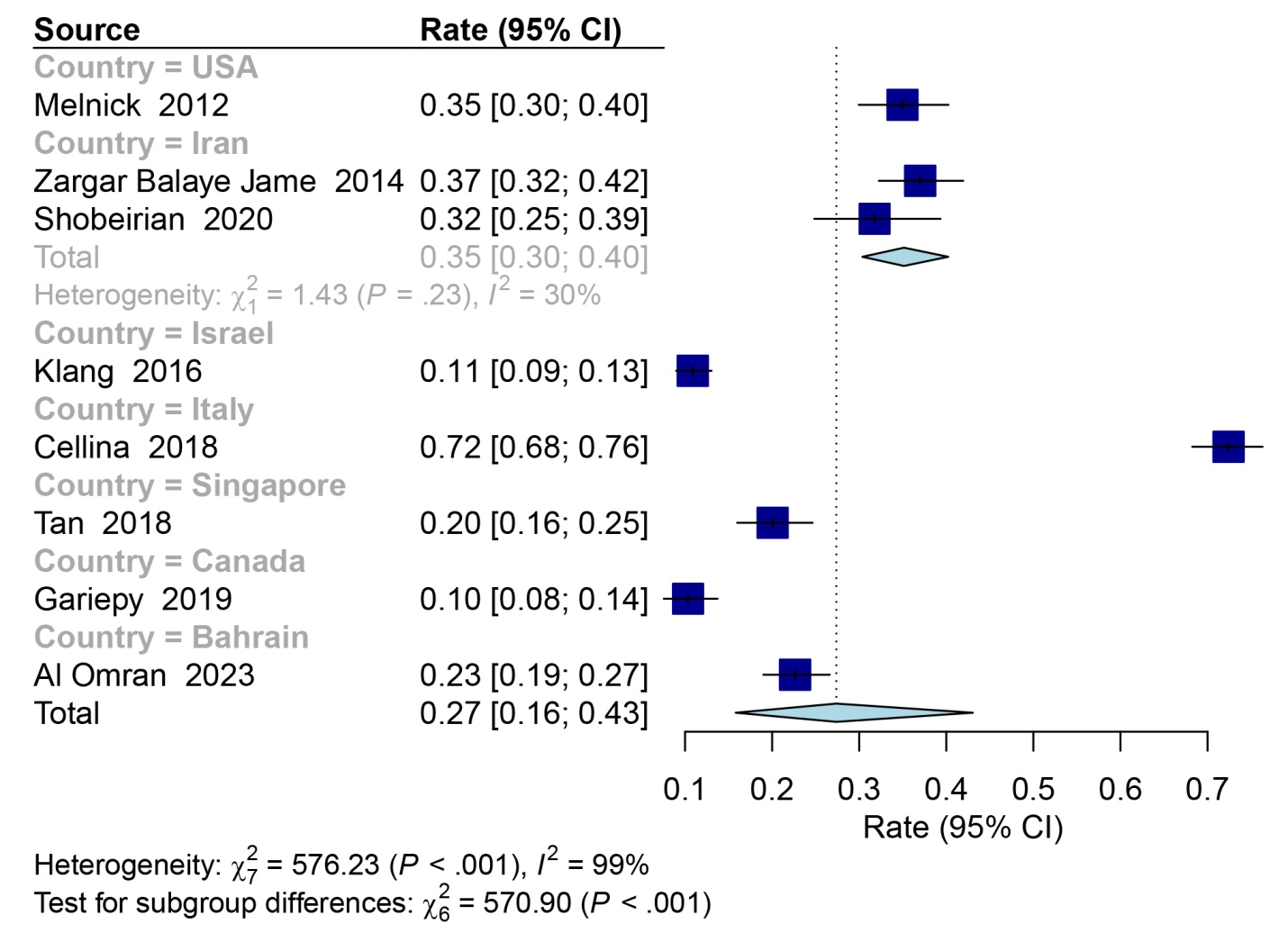


**S1 Fig 6.**

Supplement: S6 Fig — Random effects model used for analysis (95% confidence interval). The rate of CT Overuse of scan in patients with mild head injury was 35% [95% CI: 30:40] in America, 35% [95% CI: 30:40] in Iran, 23% [95% CI: 19:27] in Bahrain, 20% [95% CI: 16:25] in Singapore, 11% [95% CI: 9:13] in Israel and 10% [95% CI: 8:14] in Canada. (DOCX) [file pone.0293558.s006.docx]
