# Supplementary material for: Overuse of computed tomography for mild head injury: A systematic review and meta-analysis
Source: PLoS One. 2024 Jan 11;19(1):e0293558. doi: 10.1371/journal.pone.0293558 (PMC10783716; doi:10.1371/journal.pone.0293558)
Supplement: S2 Table — (DOCX) [file pone.0293558.s008.docx]

| **ABSTRACT** | | | **Location where item is reported** |
| --- | --- | --- | --- |
| **Title** | | | |
| Title | 1 | Identify the report as a systematic review. | Title , Page 2 |
| **Background** | | | |
| Objectives | 2 | Provide an explicit statement of the main objective(s) or question(s) the review addresses. | Background , Page 2 |
| **Methods** | | | |
| Eligibility criteria | 3 | Specify the inclusion and exclusion criteria for the review. | Methods , Page 2 |
| Information sources | 4 | Specify the information sources (e.g. databases, registers) used to identify studies and the date when each was last searched. | Methods, Page 2 |
| Risk of bias | 5 | Specify the methods used to assess risk of bias in the included studies. | Methods, Page 2 |
| Synthesis of results | 6 | Specify the methods used to present and synthesise results. | Methods, Page 2 |
| **Results** | | | |
| Included studies | 7 | Give the total number of included studies and participants and summarise relevant characteristics of studies. | Results , Page 2 ,3 |
| Synthesis of results | 8 | Present results for main outcomes, preferably indicating the number of included studies and participants for each. If meta-analysis was done, report the summary estimate and confidence/credible interval. If comparing groups, indicate the direction of the effect (i.e. which group is favoured). | Results, Page 2 ,3 |
| **Discussion** | | | |
| Limitations of evidence | 9 | Provide a brief summary of the limitations of the evidence included in the review (e.g. study risk of bias, inconsistency and imprecision). | Conclusion , Page 3 |
| Interpretation | 10 | Provide a general interpretation of the results and important implications | Conclusion, Page 3 |
| **Other** | | | |
| Funding | 11 | Specify the primary source of funding for the review. | Other |
| Registration | 12 | Provide the register name and registration number. | Main text , Page 14 |

**S1 Table 2.**
