# Supplementary material for: Overuse of computed tomography for mild head injury: A systematic review and meta-analysis
Source: PLoS One. 2024 Jan 11;19(1):e0293558. doi: 10.1371/journal.pone.0293558 (PMC10783716; doi:10.1371/journal.pone.0293558)
Supplement: S3 Table — (DOCX) [file pone.0293558.s009.docx]

**Search strategy**

| **Database** | **Search strategy** |
| --- | --- |
| **Scopus** | ( ( TITLE-ABS-KEY ( brain AND concussion ) OR TITLE-ABS-KEY ( neurocognitive AND disorders ) OR TITLE-ABS-KEY ( mild AND traumatic AND brain ) ) ) AND ( ( TITLE-ABS-KEY ( medical AND overuse ) OR TITLE-ABS-KEY ( overmedicalization ) OR TITLE-ABS-KEY ( overmedicalisation ) OR TITLE-ABS-KEY ( overtreatment ) OR TITLE-ABS-KEY ( over-treatment ) OR TITLE-ABS-KEY ( overuse ) OR TITLE-ABS-KEY ( unnecessary ) OR TITLE-ABS-KEY ( unwarranted ) OR TITLE-ABS-KEY ( inappropriate ) OR TITLE-ABS-KEY ( deprescribing ) OR TITLE-ABS-KEY ( de-implementation ) OR TITLE-ABS-KEY ( deimplementation ) ) ) |
| **Pubmed** | ((("Medical Overuse"[Mesh] OR Overmedicalization[tiab] OR Overmedicalisation[tiab] OR Overtreatment[tiab] OR "Over-treatment"[tiab] OR ((Overuse[tiab] OR Unnecessary[ti] OR Unwarranted[tiab] OR Inappropriate[ti] OR Deprescribing[tiab] OR De-implementation[tiab] OR Deimplementation[tiab]))) AND (((Brain Concussion[Title/Abstract]) OR (Neurocognitive Disorders[Title/Abstract])) OR (mild traumatic brain[Title/Abstract])) |
| **Web of Sciences** | **((((((((((((((TI=(Medical Overuse)) OR TI=(Overmedicalization)) OR TI=(Overmedicalisation)) OR TI=(Overtreatment)) OR TI=(Over-treatment)) OR TI=(Overuse)) OR TI=(Unnecessary)) OR TI=(Unwarranted)) OR TI=(Inappropriate)) OR TI=(Deprescribing)) OR TI=(De-implementation)) OR TI=(Deimplementation)) AND TI=(mild traumatic brain)) OR TI=(Brain Concussion)) OR TI=(Neurocognitive Disorders)** |
| **Embase** | **('Medical Overuse':ab,ti OR 'Overmedicalization':ab,ti OR 'Overmedicalisation':ab,ti OR 'Overtreatment ':ab,ti OR 'Overuse':ab,ti OR 'Unnecessary':ab,ti OR 'Unwarranted':ab,ti OR 'Inappropriate':ab,ti OR 'Deprescribing':ab,ti OR 'De-implementation':ab,ti OR 'Deimplementation':ab,ti) AND ('mild traumatic brain':ab,ti OR 'Brain Concussion':ab,ti OR ' Neurocognitive Disorders':ab,ti)** |

**S1 Table 3.**
