# Supplementary material for: Overuse of computed tomography for mild head injury: A systematic review and meta-analysis
Source: PLoS One. 2024 Jan 11;19(1):e0293558. doi: 10.1371/journal.pone.0293558 (PMC10783716; doi:10.1371/journal.pone.0293558)
Supplement: S4 Table — (DOCX) [file pone.0293558.s010.docx]

| **Study (Reference)** | **Year** | **Selectin** | | | | **Comparability based on design and analysis (2)** | **Outcome** | | **Total** |
| --- | --- | --- | --- | --- | --- | --- | --- | --- | --- |
|  |  | **RS (1)** | **SS (1)** | **NR (1)** | **ACS (1)** |  | **AO (2)** | **ST (1)** |  |
| Melnick [20] | 2012 | 1 | 1 | 1 | 1 | 2 | 2 | 1 | 9 |
| Zargar Balaye Jame [21] | 2014 | 1 | 1 | 1 | 0 | 1 | 2 | 1 | 7 |
| Klang [22] | 2016 | 1 | 1 | 1 | 1 | 1 | 2 | 0 | 7 |
| Cellina [23] | 2018 | 1 | 1 | 1 | 1 | 1 | 2 | 1 | 8 |
| Tan [24] | 2018 | 1 | 1 | 0 | 1 | 1 | 1 | 1 | 6 |
| Gariepy [25] | 2019 | 1 | 1 | 1 | 1 | 1 | 2 | 1 | 8 |
| Shobeirin [26] | 2020 | 1 | 1 | 2 | 2 | 2 | 1 | 1 | 8 |
| AlOmran [27] | 2023 | 1 | 1 | 1 | 1 | 2 | 1 | 1 | 8 |

**Selection: (RS:** Representativeness of the sample**, SS:** Sample size**, NR:** Non-respondents**, AE:** Ascertainment of the exposure**), Outcome: (AO:** Assessment of the outcome, **ST:** Statistical test).

### S1 Table 4.
