# Supplementary material for: Overuse of computed tomography for mild head injury: A systematic review and meta-analysis
Source: PLoS One. 2024 Jan 11;19(1):e0293558. doi: 10.1371/journal.pone.0293558 (PMC10783716; doi:10.1371/journal.pone.0293558)
Supplement: Attachment — Submitted filename: Review.docx [file pone.0293558.s011.docx]

The manuscript entitle “Overuse of computed tomography for mild head injury: a systematic review and meta-analysis” was interesting and showed a high rate of using CT for a mild head injury, hence it might be useful for policymakers (developing guideline). However; many revisions should be done, particularly in the method and the result parts.

**Title: -**

**Abstract**

1. Write it according to PRISMA 2020 for Abstracts Checklist (many revision are needed)
2. Apply the other comments of text, if applicable**.**

**Keywords**

1. Write it based on MeSH.

**Introduction**

1. This part was lengthy and needs some revision.

**Introduction, 2^nd^ paragraph**

1. The authors wrote about the management and consequence of the trauma. While the main aim of the study was the diagnosis. Delete it.

**Introduction, 3^rd^ and 4^th^ paragraphs**

1. Mix these paragraphs and summarize them.

**Introduction, 5^th^ paragraphs**

1. “as reported in studies conducted across different countries” in the aim part: I think this issue was not the main aim of your studies. Indeed, there were no subgroups regarding the region.

**Method**

1. This part needs many revisions and responses. See the comments, please.
2. Certainty (quality) of evidence should conduct.
3. A table of findings should be added.

**Protocol and Registration**

1. Any deviation should mention here. For example: review question#2: “What is the pooled prevalence of computed tomography overuse for mild head injury based on region?” I did not find any subgroup regarding the region.

**Study Design:**

1. I could not find the design of study in this paragraph.
2. For PRISMA 2020 Checklist, you should report the page in the checklist in the supplementary file.
3. Add the PRISMA 2020 for Abstracts Checklist in the supplementary file too.

**Search Strategy and Selection Criteria**

1. Why did the author set a time limitation (January 2000)?
2. Add the page of the appendix.
3. “two team”. Add the initial name of the researchers.
4. “to find additional studies”. Delete it.
5. "studies were published in peer-reviewed journals" was one of the inclusion criteria. Please explain that, while you should have considered the published article, you searched gray literature. More details should be added around gray literature.

**Eligibility Criteria**

1. When some data were missed/ full texts were unavailable, it should be supposed to contact the corresponding author. Please explain why the author did not contact the corresponding authors.
2. “those published as reviews”. Delete it. Previously it was mentioned that the observational study designs were included.
3. It is unclear whether the authors consider or not considered unpublished records.
4. “intervention studies, or studies in languages other than English.” The type and language limitations were mentioned, previously.

**Data Collection**

1. “two author teams extracted information”. Add the initial name of the researchers.
2. Gender ratio or number?
3. “criteria for CT scan performance, and prevalence of overuse”. I could not find these variables in the table.
4. “resolved by a senior researcher”. Add the initial name of the researchers.
5. “a third reviewer was introduced”. Add the initial name of the researcher.
6. “designed by the authors' group”. All authors or some of them (if some, add the initial name of them).

**Risk of Bias of Individual Studies**

1. How many authors did that? Add the initial name of them.

**Statistical Analysis**

1. "inverse variance" and "Dersimonian-Laird" are two different models. Why did the authors use two different computations?
2. Why you did not conduct a sensitivity analysis by omitting high RoB studies? What was the benefit of conducting a RoB assessment?
3. mean of age
4. I recommend conducting quantitative publication measurement (Regression-based Egger/Harbord/Peters test). In addition, funnel plots could be add in supplementary file.

**Results**

1. Many revisions are needed.

**Study Selection**

1. “and 45 were excluded.” According to PRISMA checklist 2020, 16b: Cite studies that might appear to meet the inclusion criteria, but which were excluded, and explain why they were excluded. Hence, cite the articles and explain the reason for the exclusion in a table in the supplementary file.
2. “Figure 1”. Use PRISMA 2020 flow diagram for new systematic.

**Study Characteristics**

1. “Out of a total of 8 articles, two were done in Iran. The rest of the countries had assigned one article each, and”. Delete it.
2. Add more details regard the “criteria for CT scan performance” and “criteria for diagnosing acute head injury”.

**Risk of Bias**

1. The visualization cloud makes this part easier for the reader.
2. More details are needed: for example, which domain was high risk among the studies? Add results for each study and each domain.
3. “The risk of bias of the included studies was assessed through the use of the NOS checklist.” Delete it.
4. “One study received a score of 6, two studies scored 7, four studies scored 8,” Cite the studies.

**Synthesis of Results**

1. “using a random model”. Delete it.
2. Write the I2 in the bracket and delete “This was observed with I^2^=99%”. Hence, the correct from is [95% CI: 16-43; I^2^=99%].
3. “Figure 2”. Delete this figure. Figure 3 was enough.
4. “To ensure the robustness of the sensitivity analysis, the impact of each study was consistent before and after the analysis. Furthermore, the effect of study order was examined through cumulative meta-analysis.” Here, you should report the result here.
5. “Here, you should report the result here”. In the method part, any subgroup should be mentioned.
6. Add I^2^ when reporting 95% CI.
7. “Accordance clinical decision rules”. In the method part, any subgroup should be mentioned. These variables are also not reported in data extraction table 1.

**Comparison of criteria used in included studies**

1. This part was not clear. In addition, neither declare in the aim nor method part. I think it should be deleted.

**Meta-regression (last paragraph of result)**

1. “This part was not clear. In addition, neither declare in the aim nor method part. I think it should be deleted.” Delete it.
2. “The results are presented in Table 2”. The results of meta-regression are presented in Table 2.

**Discussion**

1. Some parts of the discussion should reassess after the method and result revisions.

**Discussion, 1^st^ paragraph**

1. “However, the rate range reported in the 8 studies included in the final analysis varied.” Delete it
2. “It is important to note that differences in study populations, including age, gender, comorbidities, and severity of head injury, may have influenced the rate of overuse observed in our study”. Some of these variables were available in your studies and could be checked (or checked) through mete-regression to support your discussion.
3. “Furthermore, our study found that the rate of overuse of CT scans varied between countries,” you could do a subgroup based on country to support your discussion.

**Discussion, 2^nd^ paragraph**

1. “Glasgow Coma Scale (GCS)”. Delete “Glasgow Coma Scale”. Previously you define this abbreviation.

**Discussion, 3^rd^ paragraph: -**

**Discussion, 4^th^ paragraph**

1. It needs to reassess after revision or the author's response.

**Discussion, 5^th^ paragraph**

1. First line, “our study found that”. Delete it.

**Discussion, 6^th^ paragraph: -**

**Discussion, 7^th^ paragraph**

1. This part (policies were suggested) was a good point of this study.

**Strengths and Limitations**

1. Add more strengths regarding the work.
2. How did the authors deal with heterogeneity?

**Conclusion**

1. “Based on the findings of our meta-analysis, it can be concluded that”. Delete it.
2. “for mild head injury patients”. Delete it.
3. “Future research should aim to identify barriers and facilitators to the implementation of such guidelines, and to evaluate the impact of interventions aimed at reducing excessive CT scan use in mild head injury patients.” Delete it.

**Data Availability**

1. Data relevant to the meta-analysis were not in the supplementary file.

**Abbreviations**

1. “CI: confidence intervals”. Write it as “CI: Confidence intervals”. Check all words.

**Funding**

1. “The author(s) received no financial support for the research, authorship, and/or publication of this article.” In CRD, Lorestan University of Medical Sciences was mentioned for funding. Transparency is very important for each study.

**Author Contributions**

1. Some authors were added to the paper while their names were not in the CRD protocol. I recommend, if any deviation from protocol was done, revise the protocol in CRD.
2. While the authors declare "no funding", what were they mean by mentioning resources?

**References:-**

**Table 1**

1. In “First author” column: Delete the initial names.
2. “Glasgow coma scale”. Write it “Glasgow Coma Scale”
3. “Age”. Add the value of this variable.
4. “NA”. Define the NA in the footnote.
5. “the Pediatric Emergency Care Applied Research Network (*PECARN*)” in footnote: write as “*PECARN*: the Pediatric Emergency Care Applied Research Network”

**Table 2: -**

**Figure 1**

1. Use PRISMA 2020 flow diagram.
2. What was the difference between the two last lines in excluded box?

**Figure 2**

1. Delete this figure. Figure 3 was enough.

**Figure 3.**

1. Delete the initial names.

**Figure 4, 5 and 6**

1. As previously mentioned, this part (Comparison of criteria used in included studies) was not clear.

**Supporting Information**

**Search strategy**

1. Add *“PRESS Guideline* — Search Submission & Peer Review Assessment”

**PRISMA Checklist**

1. For PRISMA 2020 Checklist, you should report the page in the checklist in the supplementary file.
2. Add the PRISMA 2020 for Abstracts Checklist in the supplementary file too.
