# Supplementary material for: Overuse of computed tomography for mild head injury: A systematic review and meta-analysis
Source: PLoS One. 2024 Jan 11;19(1):e0293558. doi: 10.1371/journal.pone.0293558 (PMC10783716; doi:10.1371/journal.pone.0293558)
Supplement: Attachment — Submitted filename: Response to the reviewers.docx [file pone.0293558.s013.docx]

**Dear editor**

I wanted to take a moment to express my sincere gratitude for your valuable comments and feedback on my manuscript for the journal. Your thoughtful and constructive input has helped to significantly improve the quality and clarity of the manuscript. I appreciate the time and effort that you put into reviewing the manuscript and providing such insightful comments. As you may be aware, we have carefully considered your feedback and have provided responses to each of the comments raised by the reviewers. Your expertise and attention to detail have been invaluable in helping to shape the final version of the manuscript. We hope that the revised manuscript meets the required standards and addresses all of your concerns.

Thank you once again for your dedication to the peer review process and for your contributions to the advancement of scientific knowledge.

Sincerely,

Dr. Masoud Behzadifar

=======================

Additional Editor Comments:

Please take note of the following corrections:

Please add sensitivity test, Baujat test and related plots. Thank you so much for your comment. We added Baujat test and sensitivity test and related plots (Figure 4 and Figure 5).
