# Supplementary material for: Overuse of computed tomography for mild head injury: A systematic review and meta-analysis
Source: PLoS One. 2024 Jan 11;19(1):e0293558. doi: 10.1371/journal.pone.0293558 (PMC10783716; doi:10.1371/journal.pone.0293558)
Supplement: Attachment — Submitted filename: Response to the reviewers-4.docx [file pone.0293558.s015.docx]

Dear Dr. Jafar Kolahi

Academic Editor of the PLOS ONE

I would like to express my sincere gratitude for your time and valuable feedback on my manuscript submitted to PLOS ONE. I appreciate the thorough review of my work and the opportunity to revise it in accordance with your comments and suggestions. I have carefully considered of your comment and have made the necessary revisions to improve the quality and clarity of the manuscript. Below, I address each of your points and provide an explanation of the changes made. I believe that the revisions have significantly strengthened the manuscript and have addressed the concerns raised during the initial review. Additionally, I have taken care to ensure that the manuscript complies with the journal's guidelines and formatting requirements. Please find attached the revised manuscript, along with a marked-up version highlighting the changes made. I hope that you will find these revisions satisfactory, and they meet the standards of the PLOS ONE Journal.

With best wishes

Masoud Behzadifar

Correspondence author

**********************************************

Additional Editor Comments:

Please extend figure legends.

Thank you for your constructive feedback and your valuable suggestion to extend the figure legends in our manuscript. In response to your comment, we have revised the figure legends for all figures in the manuscript. We have expanded upon the information provided in the legends to provide more context and explanation of the key findings and elements depicted in each figure. These extended figure legends now provide a more comprehensive description of the data, methodology, and results associated with each figure.
